# Alpha synuclein aggregation drives ferroptosis: an interplay of iron, calcium and lipid peroxidation

**DOI:** 10.1038/s41418-020-0542-z

**Published:** 2020-04-27

**Authors:** Plamena R. Angelova, Minee L. Choi, Alexey V. Berezhnov, Mathew H. Horrocks, Craig D. Hughes, Suman De, Margarida Rodrigues, Ratsuda Yapom, Daniel Little, Karamjit S. Dolt, Tilo Kunath, Michael J. Devine, Paul Gissen, Mikhail S. Shchepinov, Sergiy Sylantyev, Evgeny V. Pavlov, David Klenerman, Andrey Y. Abramov, Sonia Gandhi

**Affiliations:** 1 UCL Queen Square Institute of Neurology, Queen Square, London, WC1N 3BG UK; 2 The Francis Crick Institute, London, UK; 3 Institute of Cell Biophysics, Russian Academy of Sciences, Pushchino, 142290 Russia; 4 Orel State University, Orel, Russia; 5 EaStCHEM School of Chemistry, University of Edinburgh, Edinburgh, UK; 6 Department of Chemistry, University of Cambridge, Cambridge, UK; 7 Dementia Research institute at University of Cambridge, Cambridge, UK; 8 MRC Centre for Regenerative Medicine, The University of Edinburgh, Edinburgh, UK; 9 MRC Laboratory for Molecular Cell Biology, University College London, Gower Street, London, UK; 10 NIHR Great Ormond Street Hospital Biomedical Research Centre, London, UK; 11 Retrotope Inc., Los Altos, CA 94022 USA; 12 Rowett Institute, University of Aberdeen, Ashgrove Rd West, Aberdeen, AB25 2ZD UK; 13 College of Dentistry, New York University, New York, NY USA

**Keywords:** Neural ageing, Neurological disorders

## Abstract

Protein aggregation and abnormal lipid homeostasis are both implicated in neurodegeneration through unknown mechanisms. Here we demonstrate that aggregate-membrane interaction is critical to induce a form of cell death called ferroptosis. Importantly, the aggregate-membrane interaction that drives ferroptosis depends both on the conformational structure of the aggregate, as well as the oxidation state of the lipid membrane. We generated human stem cell-derived models of synucleinopathy, characterized by the intracellular formation of α-synuclein aggregates that bind to membranes. In human iPSC-derived neurons with SNCA triplication, physiological concentrations of glutamate and dopamine induce abnormal calcium signaling owing to the incorporation of excess α-synuclein oligomers into membranes, leading to altered membrane conductance and abnormal calcium influx. α-synuclein oligomers further induce lipid peroxidation. Targeted inhibition of lipid peroxidation prevents the aggregate-membrane interaction, abolishes aberrant calcium fluxes, and restores physiological calcium signaling. Inhibition of lipid peroxidation, and reduction of iron-dependent accumulation of free radicals, further prevents oligomer-induced toxicity in human neurons. In summary, we report that peroxidation of polyunsaturated fatty acids underlies the incorporation of β-sheet-rich aggregates into the membranes, and that additionally induces neuronal death. This suggests a role for ferroptosis in Parkinson’s disease, and highlights a new mechanism by which lipid peroxidation causes cell death.

## Introduction

Synucleinopathies are neurodegenerative diseases that are characterized by the abnormal aggregation of the protein α-synuclein, and include Dementia with Lewy Body disease, Parkinson’s disease (PD), and multiple system atrophy [1]. Within this group of diseases, it is evident that α-synuclein aggregation, or Lewy Body pathology, occurs in diverse cell groups including the enteric nervous system, midbrain dopaminergic neurons and cortical neurons [2–5]. Genetic evidence shows that alteration in the concentration or structure of α-synuclein causes the synucleinopathies, in particular PD. Missense mutations, and duplications or triplications of the SNCA gene, lead to autosomal dominant PD [6–9] that is indistinguishable from sporadic PD, apart from early onset and a more aggressive course. Variations in the SNCA gene that lead to increased α-synuclein expression represent a genetic risk factor for sporadic PD [10].

Compelling pathological and genetic data defines α-synuclein as the cause of synucleinopathies, and raises the question of how aggregation induces cellular dysfunction and death. During aggregation, α-synuclein transitions from an intrinsically disordered monomeric protein to form small soluble oligomers with increasing β-sheet content, followed by protofibrils and insoluble fibrils. The soluble intermediate oligomeric species of α-synuclein may be ‘toxic’ to cells, and the toxicity of certain oligomers of α-synuclein may be attributed to specific structural characteristics that confer damaging properties [11]. It is well established that α-synuclein interacts with lipids in its monomeric form, where it may regulate synaptic vesicle trafficking [12]. Importantly, oligomers of high β-sheet content and exposed hydrophobic residues also interact with lipids, and disrupt or damage membrane structure, resulting in aberrant ion fluxes [13–15]. We have also demonstrated that oligomeric species can generate reactive oxygen species within the cell, and this leads to the oxidation of lipids in the plasmalemmal and mitochondrial membranes, as well as oxidation of mitochondrial proteins [13, 16, 17]. Oligomer-induced oxidation events open the mitochondrial permeability transition pore, leading to apoptosis [18]. However, other forms of cell death also exist, and death by ‘ferroptosis’ has emerged to describe cell toxicity driven by the iron-dependent accumulation of lipid peroxides [19].

In this study, we utilized two human stem cell-derived models of synucleinopathy to investigate how protein aggregation, calcium signaling, and redox biology interact to induce toxicity. We investigate (i) the effect of cellular uptake of exogenously applied recombinant oligomers, in which we can control the structure and concentration of the species and (ii) the effect of longer term endogenous increased expression of α-synuclein from SNCA mutations, and therefore the consequence of endogenously generated oligomeric species. α-synuclein aggregation occurs in human neurons, and these aggregates deregulate physiological calcium signaling, an effect dependent on the interaction between aggregates and membranes. Importantly modulation of the oxidation state of the lipids changes the membrane—α-synuclein interactions, and cell viability, highlighting the cell death pathway, ferroptosis, in these models.

## Material and methods

### Human stem cell-derived models

#### Human induced pluripotent stem cell (iPSC) culture

iPSCs were derived from donors who had given signed informed consent for derivation of iPSC lines from skin biopsies as part of the EU IMI-funded program StemBANCC. All experimental protocols had approval from the London—Hampstead Research Ethics Committee (ref: 13/13/LO/0171, IRAS project ID: 100318) and R&D approval from the University College London Great Ormond Street Institute of Child Health and Great Ormond Street Hospital Join Research Office.

iPSC-derived cortical neurons from three SNCA triplication (SNCA x3) clones and three control clones were generated using standard protocols, and all experiments were performed on a minimum of three independent inductions. Experiments were repeated using the SNCA x3 clones, and an isogenic clone generated from the same patient. iPSCs were generated from a patient with early onset autosomal dominant PD owing to a triplication of the SNCA gene (encoding α-synuclein) using viral transduction of OCT4, SOX2, KLF4 and c-MYC [20]. SNCA x3 results in four copies of the SNCA gene, and a doubling of SNCA mRNA and α-synuclein protein. CRISPR/Cas9 nickase technology was employed to remove two SNCA alleles to restore the SNCA gene dosage to two copies, whilst retaining the rest of the triplication locus (that is, the isogenic control, [21]. The isogenic cell line was generated from a *SNCA x3* iPSC clone by CRISPR/Cas9 double nickase gene editing to knockout two *SNCA* alleles, reducing the allele dosage from four (in the triplication cells) to two (normal). This method retains the rest of the triplication locus intact, and therefore provides the ideal control for the effects of *SNCA* x3 alone.

iPSCs were cultured on Geltrex (Thermo-Fisher) in Essential eight medium (Thermo-Fisher) and passaged using 0.5 mm EDTA (Thermo-Fisher). Neural induction was performed through dual SMAD inhibition using SB431542 (10 µm, Tocris) and dorsomorphin dihydrochloride (1 µm Tocris) within N2B27 media—DMEM;F12 + glutamax, neurobasal, B28, N2, glutamax, insulin, non-essential amino acids, 2-mercaptoethanol, Pen/strep- (modified from ref. [22]). Cells were first passaged with dispase (Thermo-Fisher, 1:2) at day 10 upon first appearance of the neuroepithelial sheet. Upon appearance of neural rosettes at day 20–21, cells are passaged again with dispase. Cells were passaged approximately three more times before being used at day 70–90. All lines were mycoplasma tested (all negative) and performed with short tandem repeat profiling (all matched) by the Francis Crick Institute Cell service team.

#### Human embryonic stem (ES) cells culture

The hESC line was kindly provided by Dr. David Hay (University of Edinburgh), upon MRC Steering Committee approval (ref. no. SCSC11-60). The line was established at the Centre for Stem Cell Biology (University of Sheffield) under a license from the Human Fertilization and Embryology Authority, and has been validated to show the standard hESC characteristics including a normal karyotype. In brief, pCAG-SNCA-IRES-Venus or the control pCAG-IV were transfected into hES cells followed by antibiotic selection to allow the generation of clones with stable expression of SNCA. Clones exhibiting normal morphology, growth and differentiation behavior were selected and characterized for SNCA expression, and two clones with near normal levels of SNCA expression (here designated control) and high levels of SNCA expression (designated as hES OE syn) were utilized for further studies.

For neural induction, hES cells were dissociated into single cells with Accutase (Gibco, Cat. no. A11105-01) and plated on a Matrigel-coated six-well plate in mTeSR1 medium. Cells were fed daily until they reached 90% confluency or above. Neural induction started at day 0, when mTeSR1 was replaced with hESC medium lacking FGF2, supplemented with 10 μm SB431542 (Tocris) and 100 nm LDN-193189 (Stemgent). Cells were fed daily with this medium until day 4. From day 5 to day 11, SB431542 was withdrawn and cells were fed every other day with a mixture of hESC medium and N2B27, which was gradually added into culture medium from 25%, 50%, 75%, and 100% at day 5, day 7, day 9, and day 11, respectively. pCAG-SNCA-IRES-Venus or the control pCAG-IV were transfected into hES cells followed by antibiotic selection to allow the generation of clones with stable expression of SNCA. Clones exhibiting normal morphology, growth and differentiation behavior were selected and characterized for SNCA expression, and two clones with near normal levels of SNCA expression (here designated control) and high levels of SNCA expression (designated as hES OE syn) were utilized for further studies.

### Aggregation of α-synuclein

Wild-type α-synuclein and A90C variant were purified from *Escherichia* *coli* as previously described by Hoyer et al. [23]. All α-synuclein aggregations (using labeled or unlabeled protein) were conducted in LoBind microcentrifuge tubes (Eppendorf) to limit surface adsorption.

For the aggregation reactions of unlabeled recombinant α-synuclein, a 70 μm solution of wild-type α-synuclein in 25 mm Tris buffer with 100 mm NaCl pH 7.4 (supplemented with 0.01% NaN_3_ to prevent bacterial growth during aggregation) was incubated at 37 °C with constant agitation at 200 rpm (New Brunswick Scientific Innova 43), during which time aliquots were taken.

For the aggregation reactions of labeled α-synuclein, the A90C variant of monomeric α-synuclein was labeled with maleimide-linked Alexa Fluor 488 (AF488) or Alexa Fluor 594 (AF594) (Life Technologies) as described previously [16, 24]. The excess dye was removed by passing the labeled protein through a P10 desalting column containing Sephadex G25 matrix (GE Healthcare, Waukesha, WI). After elution, protein concentration was determined using a nanodrop and the sample divided into aliquots, before being flash-frozen in liquid nitrogen and stored at −80 °C. Each aliquot was only thawed once prior to use. AF488-labeled and AF594-labeled monomeric α-synuclein were diluted in Tris buffer (same composition as mentioned above) at a concentration of 70 µm. For FRET experiments, AF488-labeled and AF594-labeled monomeric α-synuclein were incubated together to a final concentration of 70 µm. Similar to the aggregation of unlabeled α-synuclein, the reaction was performed in the dark at 37 °C with constant agitation at 200 rpm (same incubator as above) and aliquots were withdrawn at specific time points.

Different time points of unlabeled α-synuclein aggregation reaction were characterized using a highly sensitive single-molecule method termed SAVE (Single Aggregate Visualization by Enhancement) imaging, which uses single-molecule fluorescence microscopy to detect the benzothiazole salt thioflavin-T (ThT) (Supplementary Fig. 1). Upon binding to β-sheet structures, ThT fluorescence increases allowing individual aggregated species to be detected. From 2 h onwards, the number of diffraction limited fluorescent puncta increases, which represents the emergence of oligomers and at later time points (>24 h), fibrils as long as 5 µm are observed. For this study, we used time point 0 h (no aggregates), 8 h (maximum number of oligomers without the presence of fibrils) and 24 h [16](fibrils). The kinetics of labeled α-synuclein reaction is different from the unlabeled and the time point that maximizes the number of oligomers is around 29 h and fibrils appear after 72 h.

### Deuteration of PUFAs

Deuteration of PUFAs, deuterated polyunsaturated fatty acids (d-PUFAs) were prepared as described previously [25] and used as free acids. Non-deuterated PUFAs were obtained from Sigma-Aldrich (99%; St. Louis, MO, USA). Cells were pre-incubated with 10 μm d-PUFA in the culturing media for 48 h prior to experiment and washed with HEPES-buffered salt solution (HBSS) before experiments.

### Live Imaging (fluorescence measurements)

Fluorescent indicators (fura-2 AM, dihydroethidium, C11-BODIPY, SYTOX Green) were used to measure [Ca^2+^], reactive oxygen species (ROS), and lipid peroxidation, and cell death, respectively. The fluorescent data were collected using a cooled camera device or a confocal microscope. For fluorescence measurements with cooled camera device, data were obtained on an epifluorescence inverted microscope equipped with a ×20 fluorite objective. For confocal microscopy, images were obtained using an either Zeiss 710 or 880 (airy) vis CLSM equipped with a META detection system and a ×40 oil immersion objective. Illumination intensity was kept to a minimum (at 0.1–0.2% of laser output) to avoid phototoxicity and the pinhole set to give an optical slice of ~2 µm. All data presented were obtained from at least three coverslips and 2–3 different inductions.

For measurements of [Ca^2+^]c, cells were loaded for 30 min at room temperature with 5 µm Fura-2 AM with 0.005% pluronic acid in a HBSS composed of (mm): 156 NaCl, 3 KCl, 2MgSO_4_, 1.25 KH_2_PO_4_, 2 CaCl_2_, 10 Glucose, and 10 HEPES; pH adjusted to 7.35 with NaOH. [Ca^2+^]c was monitored in single cells using excitation light provided by a Xenon arc lamp, the beam passing through monochromator centered sequentially at 340 and 380 nm (Cairn Research, Kent, UK). Emitted fluorescence light was reflected through a 515 nm longpass filter to a cooled CCD camera (Retiga, QImaging, Canada). All imaging data were collected and analyzed using software from Andor (Belfast, UK). The Fura-2 data have not been calibrated in terms of [Ca^2+^]c because of the uncertainty arising from the use of different calibration techniques. For Dihydroethidium (Het) we generated ratios of the oxidized form (ethidium) excited at 530 nm and measured using a 560 nm longpass filter and the reduced form with excitation at 380 nm, measured between 415 and 470 nm.

To assess lipid peroxidation, cells were loaded with C11-BODIPY (581/591, 2 μM, Molecular Probes) in HEPES-buffered HBSS for 20 min prior to imaging and then excited using the 488 and 565 nm laser and fluorescence measured from 505 to 550 nm and above 580 nm (409 objective) using a confocal microscopy. The intensely fluorescent C11-BODIPY 581/591 fluorophore is an intrinsically lipophilic dye, which results in accumulation within membranes. Upon oxidation of the polyunsaturated butadienyl portion of the dye, there is a shift of the fluorescent emission peak from 590 nm to 510 nm, and it remains lipophilic, thus reflecting lipid peroxidation in membranes.

For SYTOX green (Molecular Probes), cells were loaded with SYTOX green in HEPES-buffered HBSS for 15 min. High-throughput images were acquired using an Opera Phenix High-Content Screening System (PerkinElmer). SYTOX green staining was imaged by 488 nm and 405 nm for Hoechst staining nuclei. Total 17–22 fields of images were taken per wells. Then the percentage of cell death was quantified by the ratio between the number of Sytox green-positive cells and the total number of Hoechst expressing cells per image using a Columbus StudioTM Cell Analysis Software.

To visualize cytoplasmic membrane, cells were washed with HBSS and incubated with either CellMask deep red Plasma membrane Stain (TheroFisherScientific, 5 μg/ml) for 5–10 min or CellBrite Blue (Biotium, 5 μm) for 30 min in HBSS and live-cell imaging was performed.

### Measuring α-synuclein aggregate-induced Ca^2+^ influx

To characterize aggregation in neurons derived from SNCA x3, lysates and media were collected from both control and SNCA x3. Cells were lysed mechanically without using a lysis buffer. The lysates and media were collected in an Eppendorf tube and centrifuged at 15,000 rpm for 15 min. Supernatant was collected in a fresh Eppendorf tube and kept in −80 until use.

For the membrane permeabilization assay, vesicles are prepared as previously described [26]. Using this assay, it has been previously shown α-synuclein oligomers disrupt and permeabilise membranes [27, 28]. In brief, vesicles are synthesized using Phospholipids 16:0–18:1 PC and biotinylated lipids 18:1–12:0 Biotin PC (100:1) using freeze thaw method and mean diameter is 200 nm. Vesicles of oxidized lipid was made using oxPAPC (Oxidized 1-palmitoyl-2-arachidonoyl-sn-glycero-3-phosphocholine) and 18:1–12:0 Biotin PC. Each vesicle is filled with 100 μm Cal-520 dye and immobilized in PLL-g-PEG-coated plasma cleaned glass coverslips using biotin-neutravidin linkage. The surrounding of the vesicles was filled with Ca^2+^ buffer. In all, 50 µL of sample was incubated with the vesicles for 15 min and Ca^2+^ influx was quantitatively measured using a homebuilt total internal reflection fluorescence microscope based on an inverted Nikon Ti-2 microscope. In all, 488 nm laser was focused back-focal plane of the ×100, 1.49NA oil immersion objective lens used to excite the Cal-520 dye. The fluorescence signal was collected by the same objective and magnified 1.5 times. Then the emission light was passed through a longpass filter (BLP01-488R-25) and a band pass filter (FF01-520/44-25) before imaged in EmCCD camera. To check if the aggregate present in SNCA x3 media and lysates are composed of α-synuclein, we have used previously reported method to determine the composition of the aggregates [29, 30]. Media was incubated with Anti-Alpha-synuclein (phospho S129) antibody (Abcam ab51253), for 30 min and then added to the coverslips containing dye filled vesicles. Statistical significance test was performed using two sample unpaired *t* test.

### Electrophysiology

#### Single-channel electrophysiology in living neurons

Patch-clamp recordings of α-synuclein channels overexpressed in iPSC-derived neurons were performed in perfusion solution containing: 124 mm NaCl, 3 mm KCl, 26 mm NaHCO_3_, 1.25 mm NaH_2_PO_4_, 10 mm d-glucose, 2 mm CaCl_2_, 2 mm MgCl_2_, bubbled with 95:5 O_2_/CO_2_ (pH 7.4). To isolate response of α-synuclein channels we added to external solution 50 µm D-APV, 10 µm NBQX, 100 µm picrotoxin and 1 µm strychnine. Outside-out patches were excised from cell soma and held at −70 mV membrane potential. Intrapipette solution contained 117.5 mm Cs-gluconate, 17.5 mm CsCl, 10 mm KOH-HEPES, 10 mm BAPTA, 8 mm NaCl, 5 mm QX-314, 2 mm Mg-ATP, 0.3 mm GTP. As a control, we performed recordings from outside-out patches pulled from iPSC-derived neurons received from healthy volunteers.

Recordings were performed at 33–35 °C using Multiclamp-700B amplifier, in whole-cell mode; signals were digitized at 10 kHz. Recording electrodes were pulled from the thick-wall borosilicate glass capillaries and fire-polished to 5–7 MOhm resistance.

In human stem cell-derived neurons, to calculate and visualize amplitude characteristic for ion channel openings, we constructed all-points histograms with 0.1 pA bin, and fitted them with a double-Gaussian function:

$$F = \frac{{p_1e^{ - \frac{{\left( {n - m_1} \right)^2}}{{2\sigma _1^2}}}}}{{\sigma _1\sqrt {2\pi } }} \, + \, \frac{{p_2e^{ - \frac{{\left( {n - m_2} \right)^2}}{{2\sigma _2^2}}}}}{{\sigma _2\sqrt {2\pi } }},$$

where m1 and m2 are the mode values of Gaussians, σ1 and σ2 are the standard deviations of corresponding modes, *n* is the value of electrical current, and p1 and p2 are the fitting constants.

With this approach the mode value for the open state was fitted as 2.17 pA.

#### Channel activity in liposomes

Giant liposomes were prepared as described previously [31]. In brief, liposomes were formed by sonication of lipid (type IV-S soybean l-phosphatidylcholine; Sigma-Aldrich) in water. Liposomes (600 µg lipid) were mixed with 5 mm HEPES, pH 7.4 (50 µl volume) and with protein sample (5 µl), and dotted on a glass slide. Samples were dehydrated (3 h) and rehydrated overnight with 150 mm KCl, 5 mm Hepes, pH 7.4, at 4 C. Patch-clamp procedures and analysis used were described previously [18]. Membrane patches were excised from liposomes after formation of a giga-seal using micropipettes with ~0.4 m diameter tips and resistances of 10–20 ΩM at room temperature. Voltage clamp was performed with the excised configuration of the patch-clamp technique using an Elements eONE patch-clamp amplifier in the inside-out mode. Voltages are reported as pipette potentials. Current traces were processed using pCLAMP software. Traces for figure and all-points histograms were prepared using Origin 9.

### ELISA assay

To measure the concentration of oligomeric α-synuclein, cell lysate were mechanically collected from CTRL and SNCA x3 neurons. α-synuclein oligomer was analyzed using human α-synuclein oligomer (non A4 component of amyloid precursor) ELISA kit (CSB-E18033h, Generon) and then normalized by total protein per well using Pierce BCA Protein Assay Kit (23225, ThermoFisherScientific).

### Aptamer staining

Cells were permeabilized with 0.25% Triton X 100 and blocked with 10% Normal Goat Serum for 20 min followed by another 3 h with 0.1% Triton X—100 and 10% Normal Goat Serum. Then cells were incubated overnight with 0.5 µm ATTO-425 labeled Aptamer [32]. Cells were washed three times with PBS and imaged.

### Immunohistochemistry

Cells (cultured in ibidi chamber) were fixed in 4% paraformaldehyde and permeabilized with 0.2% Triton-100. In all, 5% bovine serum albumin (BSA) was used to block non-specific binding. Cells were incubated with primary antibody for 1 hr at room temperature and washed three times with 5% BSA. Cells were incubated with secondary antibody for 1 hr at room temperature. Cells were imaged with PBS after three times wash. Hoechst was added in the second wash if required.

### Statistical analysis

Statistical analysis (unpaired two sample *t* test or one-way analysis of variance, *P* value is set at 0.05) and curve fitting were performed using Origin 2018 (Microcal Software Inc., Northampton, MA) software. Results are expressed as means ± standard error of the mean (SEM). *N* = number of inductions and *n* = number of cells, if not stated otherwise. Sample sizes for experiments were selected to capture adequate technical variation (number of cells; numbers of fields of view; number of coverslips) and biological variation (numbers of independent inductions; numbers of clones/patient line). Variance within each group was estimated using a F-statistics (sum of squares). All experiments were repeated minimum three times. All experiments were performed in a count balance manner and data were collected and analyzed without bias.

## Results

### Human iPS derived neurons with increased α-synuclein exhibit abnormal calcium signaling

**Fig. 1:**
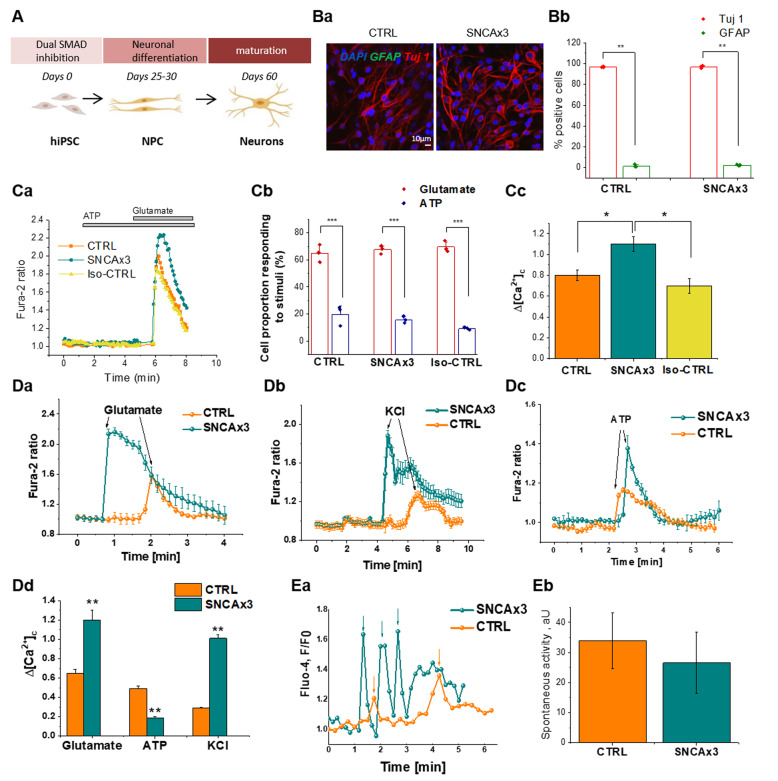
Abnormal calcium signaling in iPSC-derived neurons with SNCA x3. **a** Protocol to differentiate iPSC to cortical neurons. **b** The majority of cells are Tuj 1 (and not GFAP-positive) in both control (CTRL) and mutant (SNCA x3) lines, (a) Representative images with Tuj 1 (neuronal marker, red) and GFAP (astrocytic marker, green), DAPI (total cell number, blue). (b) Percentage cell proportion expressing Tuj 1 or GFAP, *N* = 3 CTRL & SNCA. **c** (a) Representative tracers of cytosolic calcium rise in response to 5 µm glutamate not to ATP. (b) Percent cell population responding to either 5 µm glutamate or 100 µm ATP, *N* = 3 CTRL & SNCA. (c) Application of 5 µm glutamate induced a rise in cytosolic calcium, which was significantly higher in SNCA x3 neurons compared with control neurons, *N* = 3 CTRL & SNCA. **d** (a) 5 µm glutamate induces calcium signaling. (b) Depolarization of cells with 50 mm KCl results in opening of voltage-dependent calcium channels and a cytosolic calcium signal, which was significantly higher in SNCA x3 neurons compared with control. (c) Stimulation of the P2Y receptors with 100 µm ATP induced a cytosolic calcium signal that was significantly lower in the SNCA x3 cells compared with control. (d) Histogram demonstrates the mean values of the calcium signals induced by glutamate, KCl and ATP (mean ± SEM), *N* = 3 per condition for CTRL & SNCA x3. **e** (a) Registered events of spiking activity in individual control and SNCA triplication cells (spikes are marked with arrows, orange-CTRL, teal-SNCA x3). (b) No. of events per cell*min*1000 (mean ± SEM). *n* = 180 CTRL and 125 SNCA x3 cells. *P < 0.05, **P < 0.001, ***P < 0.0001.

Differentiation of control iPSCs (control, or CTRL), SNCA triplication iPSCs (SNCA x3), and isogenic control iPSCs, into cortical neurons was performed. The SNCA x3 mutation is a gene triplication, leading to a doubling of α-synuclein protein expression [20]. We generated enriched populations of neurons from both control and SNCA x3 mutant lines, with a small proportion of glial-like cells (Fig. 1**a**, **b**). Immunocytochemistry (α-synuclein Ab MJFR1, Abcam), demonstrated elevated α-synuclein protein expression in SNCA x3 cells (Supplementary Fig. 2 Aa & b).

We investigated whether physiological calcium responses are altered by increased expression of α-synuclein. We stimulated iPSC-derived neurons with physiological concentrations of glutamate (5 µm) and KCl (50 mm), which induces opening of potential-sensitive Ca^2+^ channels that are specific for neurons. We then stimulated a calcium signal with 100 µm ATP, which induces activation of P2Y receptors [33]. There is no significant difference in the proportion of cells responding to either glutamate (neurons) or ATP stimuli (glia-like) in CTRL, SNCA x3 and Iso-CTRL (Fig. 1**c**(a, b)). Stimulation of neurons with 5 µm glutamate induces a significantly higher signal in SNCA x3 cells compared with both isogenic control and healthy control cells (CTRL: 0.65 ± 0.04, *n* = 96, SNCA x3: 1.2 ± 0.1, *n* = 99; *p* < 0.001, Fig. 1**c**c, **d**(a, d)). The physiological [Ca^2+^]_c_ response to 50 mm KCl was also higher in SNCA x3 iPSC-derived neurons (0.29 ± 0.01, *n* = 101) when compared with control neurons (1.01 ± 0.039, *n* = 114; *p* < 0.001; Fig. 1**d**(b, d)). Stimulation with 100 µM ATP leads to an increase in [Ca^2+^]_c_ in glial-like cells, but not in neurons. This [Ca^2+^]_c_ response was significantly lower in glial-like cells with SNCA x3 when compared with control cells (0.49 ± 0.03, *n* = 68 in CTRL; *p* < 0.001; Fig. 1**d**(c, d)). We also detected an impairment of intracellular calcium homeostasis in SNCA x3 showing re-distribution of Ca^2+^ stores, and depletion of ER calcium, and increase in mitochondrial calcium (Supplementary Fig. 3).

One of the major characteristics of mature neurons is their spontaneous calcium activity. Spontaneous calcium transients were observed in all iPSC-derived cortical neurons (Fig. 1**e**a), with no significant difference in the amplitude of these transients (Fig. 1**e**b) between CTRL and SNCA x3.

### Increased expression of α-synuclein in a hES model demonstrates altered calcium signaling

To test the effect specifically of *SNCA* expression in neurons, we utilized a model of transgenic human stem cell-derived neurons engineered to express *SNCA* at control or high levels [18], on an isogenic background. Both control and *SNCA* o/e cells displayed a native-like membrane potential of 61 ± 5 mV (*n* = 4), characteristic for neurons, and generated action potentials of classical shape in response to current injection. Action potentials in *SNCA* o/e cells were generated with significantly lower frequency: 4.2 ± 1.3 Hz vs. 18.7 ± 3.4 Hz in control, *P* < 0.01, *n* = 4, 7, Student’s *t* test. In all, 1 µm of tetrodotoxin added into the perfusion solution fully suppressed action potential generation, revealing the presence of voltage-gated sodium channels in the tested cells (Supplementary Fig. 4 Aa & b).

To test the calcium response to physiological stimuli, we applied glutamate (10 µm), which induces a typical calcium transient in control neurons, and SNCA o/e (Supplementary Fig. 4 Ba & d, CTRL; *n* = 47 cells, SNCA x3; *n* = 32 cells). In agreement with previous results, glutamate-induced calcium signal in SNCA o/e was significantly higher than those found in CTRL (signal rose to 1.3 ± 0.24 Fura-2 ratio compared with 0.7 ± 0.08 Fura-2 ratio, *p* < 0.05). More than 60% cells demonstrated typical response to plasma membrane depolarization with opening of voltage-gated calcium channels (Supplementary Fig. 4Bc & d). Again, SNCA o/e cells had significantly higher calcium responses to 50 mm KCl (Supplementary Fig. 4Bc & d, CTRL; 0.92 ± 0.1 Fura-2 ratio, *n* = 39, SNCA x3; compared with 0.61 ± 0.08 Fura-2 ratio in control, *n* = 38 cells; *p* < 0.05).

Application of 100 µm ATP stimulates calcium signaling via P2Y receptors, expressed predominantly in astrocytes. Approximately 40% of cells in the field demonstrated a calcium response to application of ATP, and SNCA o/e cells demonstrated a higher ATP-induced calcium signal than control cells (Supplementary Fig. 4Bb & d, CTRL; 1.21 ± 2.2 Fura-2 ratio, *n* = 37, SNCA x3; compared with control 0.97 ± 0.07 Fura-2 ratio, *n* = 47 cells; *p* < 0.05).

### Alpha-synuclein aggregates disrupt membranes and alter membrane conductance

**Fig. 2:**
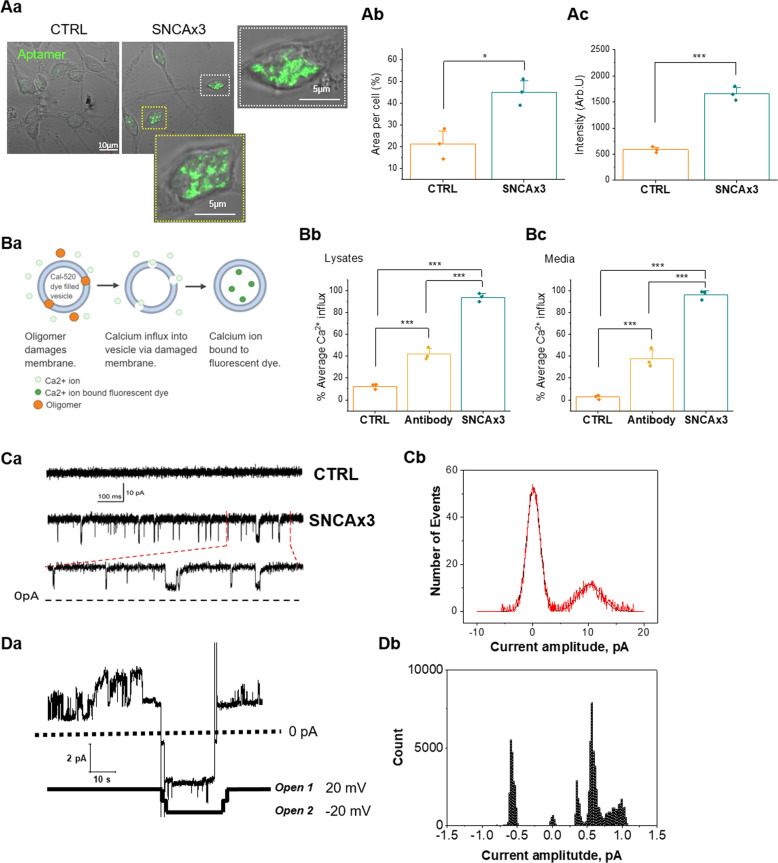
α-synuclein aggregates disrupt membranes. **a** iPSC-derived cortical neurons from patients carrying 3x SNCA exhibit aggregates. (a–c) Aptamer expression level (both area and intensity) is higher in iPSC-derived neurons with SNCA x3, *N* = 3 CTRL & SNCA x3. **b** (a) Illustration of membrane permeabilization assay by measuring Ca^2+^ influx in vesicles. (b, c) Cell lysates and media from SNCA x3 induce higher level of permeabilization than control, prevented by antibody (phospho S129) binding of α-synuclein aggregates, *N* = 3 CTRL & SNCA. **c** (a) Single-channel activity of α-synuclein in the membrane of iPSC-derived neurons. Top: control recording from outside-out patch pulled from control neurons. Middle: recording from outside-out patch pulled from SNCA x3 neurons. Bottom: extended section of the medium trace marked by the dashed lines. Vertical scale bar applying to all three traces; horizontal (time) scale bar apply to the top and medium trace. (b) All-points amplitude histogram of channel openings in the membrane of iPSC-derived neurons with SNCA x3, fitted with double-Gaussian function, *N* = 3 CTRL & SNCA. **d** (a) Single-channel activity of α-synuclein in the membrane of giant liposomes consisting of lipids. Channels open followed by partial inactivation to a stable subconductance level (open 1). After switch to –20 mV the channels open to a higher conductance state (open 2). (b) Current amplitude histogram of the α-synuclein channel activity in giant liposome patch represented in (a). *P < 0.05, **P < 0.001, ***P < 0.0001.

We investigated how α-synuclein aggregates disrupt membranes and induce ion fluxes. Using a ATTO-425-labeled Aptamer that recognizes oligomeric aggregates of α-synuclein [32], we confirmed the increase in aggregates in the SNCA x3 cells, demonstrated both by intensity, and area of cell occupied by aggregates. (Fig. 2**a**). This aptamer, using super resolution microscopy (ADPAINT) has demonstrated an increase in larger aggregates in SNCA x3 neurons [32]. We performed an oligomer ELISA to measure the soluble aggregates in the SNCA x3 and CTRL cell lysates (Supplementary Fig. 5), and demonstrated a selective increase in oligomers in the SNCA x3 neurons (Supplementary Fig. 5b).

Aggregates formed in the SNCA x3 cells, and secreted in the media, are capable of permeabilising membranes, and inducing ion fluxes. Using a single vesicle assay, we demonstrated that application of SNCA x3 lysates and media induced calcium influx across liposomes. This calcium influx was blocked after incubation of SNCA x3 lysates and media (25× dilution) with Anti- α-synuclein (phospho S129) antibody (see Fig. 2**b**a for experimental paradigm). Therefore, both secreted oligomers in the extracellular space of SNCA x3, as well as internally generated oligomers possess a structural conformation that can interact with, and disrupt, membranes (Fig. 1**b**(b, c)) to induce calcium fluxes.

To test the effect of increased endogenous α-synuclein on the membrane conductance of neurons, we performed patch-clamp recordings from outside-out membrane patches in a voltage clamp mode. To isolate the response solely of channels formed by α-synuclein aggregates, we added to the external solution 50 µm D-APV, 10 µm NBQX, 100 µm picrotoxin and 1 µm strychnine. We registered single-channel openings in patches excised from iPSC-derived neurons with SNCA x3 (channel conductance 189 ± 26 pS, *n* = 5), whereas no single-channel activity was observed in control neuronal cultures when a similar cocktail of ion channel blockers was applied (Fig. 2**c**a). However, we managed to detect activity only in a small fraction of SNCA x3 neurons (Fig. 2**c**a); five successful recordings out of 29 outside-out patches (each patch was pulled from a separate cell). To calculate the amplitude of the ion channel opening, all point histograms were constructed and fitted to a double-Gaussian function. The mode value for the open state was fitted at 2.17 pA (Fig. 2**c**b). These data suggest the presence of channel formation on the plasmalemmal membrane of neurons with high levels of α-synuclein, independent on any known ion channels.

We investigated the effect of α-syn aggregates on the ion permeability of membranes, using patch-clamp of giant liposomes exposed to recombinant α-synuclein oligomers. We detected single-channel activity induced by oligomers of α-syn, but not monomers (Fig. 2**d**(a, b)), and these channels had multiple conductance levels, with opening and partial inactivity to stable subconductance at a constant voltage, followed by higher conductance states induced at −20 mV.

### Increased expression of α-synuclein leads to generation of reactive oxygen species and membrane oxidation

**Fig. 3:**
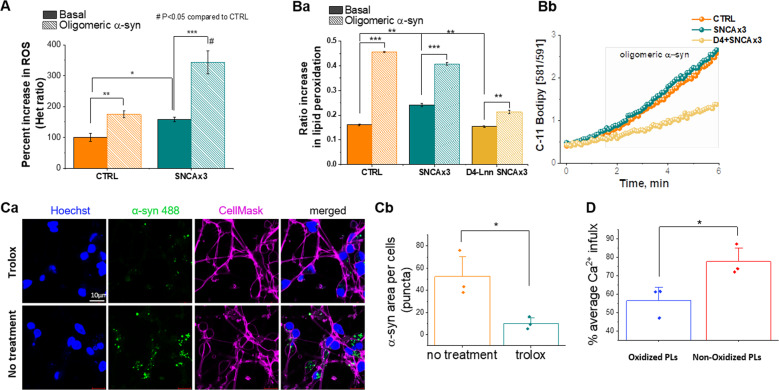
ROS production and lipid peroxidation induced by α-synuclein in neurons. **a**, **b** SNCA x3 neurons are vulnerable to exogenously applied α-synuclein oligomers. (a) Rate of ROS production in iPSC-derived SNCA x3 neurons is higher than in control neurons. (*N* = 3 CTRL, SNCA & D4-Lnn-treated SNCA). (a) Basal lipid peroxidation rate in iPSC-derived neurons with α-synuclein 3x is higher than the control neurons, but could be restored through pre-treatment with D4-Linolenic acid (D4-Lnn) in SNCA x3 neurons, which also prevented oligomer-induced lipid peroxidation (*N* = 3 CTRL, SNCA & D4-Lnn-treated SNCA). (b) Representative traces of (a). **c** Characterization of α-synuclein—membrane interactions. (a) Representative images of exogenous oligomeric α-synuclein uptake (AF488 fluorescence, green) into the cytoplasm (plasmic membranes are labeled with CellMask Depp Red dye; magenta color) in control neurons with or without Trolox treatment. Oligomer uptake was significantly reduced when membrane oxidation was inhibited by Trolox, *N* = 5 basal & Trolox. (b) Trolox inhibits uptake of aggregate forms of α-synuclein. **d** Calcium influx in the membrane permeabilisation assay was increased when vesicles composed of oxidized lipids were generated. 16:0—20:4 PC non-oxidized membrane. Ox PAPC oxidized membrane, *N* = 3 non & oxidized membrane. *P < 0.05, **P < 0.001, ***P < 0.0001.

We have reported that oligomeric α-synuclein drives the aberrant generation of intracellular superoxide and hydrogen peroxide, and the depletion of antioxidants [17, 34]. Basal superoxide production is increased in the *SNCA* *x3* iPSC-derived neurons [17] and addition of recombinant oligomers further increased superoxide production (CTRL; 175 ± 11.9 %, *N* = 4, *p* < 0.0001, SNCA x3; 158.3 % to 343.0 ± 37.3 %, *N* = 4, *p* < 0.0001, Fig. 3**a**). Consistently, hES cells with SNCA o/e also produce abnormal levels of superoxide (Supplementary Fig. 6). *SNCA x3* iPSC-derived neurons also exhibit an increase in basal lipid peroxidation from 0.161 ± 0.004 to 0.241 ± 0.007, both *N* = 4, *p* < 0.0001; addition of oligomers to control cells increased lipid peroxidation to 0.456 ± 0.003, *N* = 4, *p* < 0.0001; similarly, addition of oligomers to SNCA x3 increased lipid peroxidation to 0.408 ± 0.005, *N* = 4, *p* < 0.0001 (Fig. 3**b**).

Isotopic reinforced polyunsaturated fatty acids (d-PUFAs) incorporate into lipid membranes and render them resistant to the ROS initiated chain reaction of lipid peroxidation. We pre-treated cells with 10 µm deuterated Linolenic acid (D4-Lnn), which prevented oligomer-induced lipid peroxidation, and restored basal levels of lipid peroxidation in the *SNCA x3* cells (Fig. 3**b**). Therefore, recombinant oligomers, as well as oligomers generated in the SNCA x3 cells, induce oxidation of PUFAs, which can be modulated by the use of deuterated species.

### Lipid peroxidation influences the physical interaction of aggregates and membranes

α-synuclein- membrane interactions in cells are transient and difficult to capture. Membrane binding to the plasma membrane is a prerequisite prior to internalization of α-synuclein into the cell [35]. Labeled oligomers (oligomers generated from AF488 monomeric α-synuclein) were applied to cells loaded with either a cell mask dye, or a membrane dye. Internalization of fluorescently labeled monomer and oligomer is demonstrated in Fig. 3**c**a and Supplementary Fig. 7Aa. Vitamin E is a tocopherol that inhibits lipid peroxidation by scavenging lipid peroxyl radicals. We pre-incubated cells with Trolox, a water soluble analog of α-tocopherol which is able to incorporate into both water and lipid compartments, reduced the α-synuclein accumulation in the cell (as demonstrated by reduced AF488 area/cell, Fig. 3**c**b and Supplementary Fig. 7Ab.

Next, we tested the effect of membrane oxidation on insertion of aggregates into membranes utilizing the membrane Permeabilization assay (described in Fig. 2**c**a). Using vesicles generated from oxidized lipids, there is higher calcium influx on exposure to the same concentration of α-synuclein oligomers, compared with control lipid vesicle (Fig. 3**d**).

Together, these data suggest that under conditions of membrane oxidation, there is an enhanced membrane binding of oligomers of α-synuclein, allowing ion fluxes across the membrane, as well as entry into the cell. This interaction is reduced in cells with non-oxidized membranes.

### Lipid peroxidation drives α-synuclein-induced abnormal calcium signaling

**Fig. 4:**
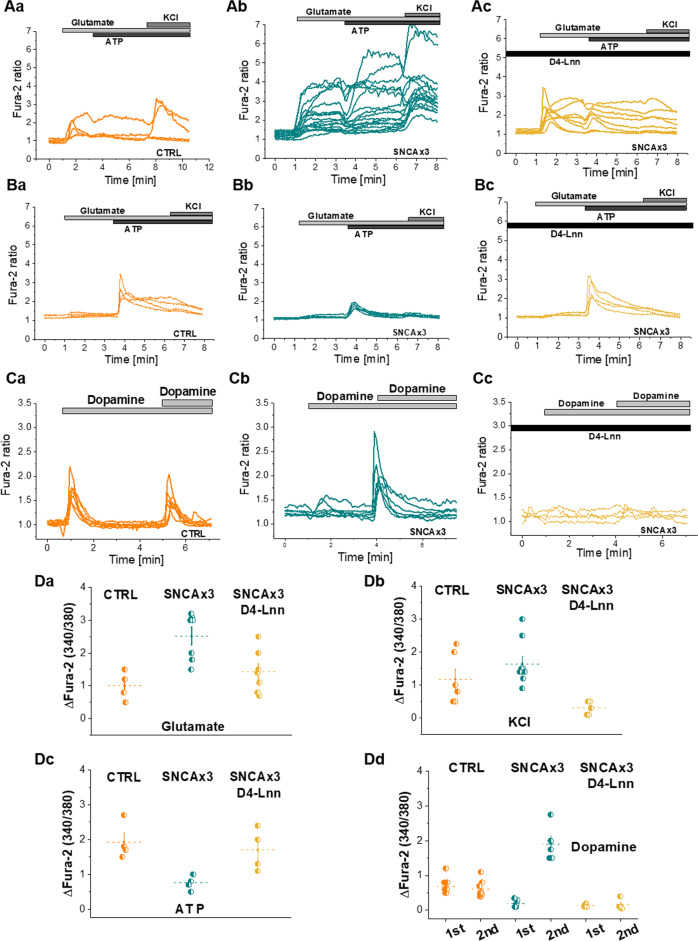
Prevention of lipid peroxidation restores calcium dysregulation in SNCA x3 neurons. **a** Representative traces showing deuterated PUFAs restore abnormal glutamate-induced calcium signaling or KCl-induced depolarization in SNCA x3 neurons (b) to control levels (c). **b** ATP-induced calcium signal is smaller in the SNCA x3 cells (b) compared with control (a). This was also restored by pre-incubation of the cells with d-PUFAs (c). **c** Double bolus of dopamine (50 µm) in SNCA x3 neurons evoked dysregulated calcium signal (b) in comparison with control response (a). (c) Pre-incubation of the SNCA x3 cells with D4-Lnn prevents dopamine-induced cytosolic calcium signals. **d** Quantification histograms depicting the preventive effect of deuterated PUFAs (D4-Lnn) on the calcium response to application of glutamate (a, *N* = 4 CTRL, *N* = 5 SNCA, *N* = 7 D4-Lnn treated SNCA), KCl (b, *N* = 5 CTRL, *N* = 7 SNCA, *N* = 4 D4-Lnn treated SNCA), ATP (c *N* = 4 CTRL, SNCA & D4-Lnn treated SNCA) or dopamine (d, *N* = 5 CTRL, *N* = 3 & 4 SNCA, *N* = 3 D4-Lnn-treated SNCA) in both control and iPSC-derived neurons with SNCA x3. *P < 0.05, **P < 0.001, ***P < 0.0001.

Next we tested whether the α-synuclein-induced calcium signaling is affected by lipid peroxidation. We demonstrated abnormal glutamate-induced calcium signaling in *SNCA x3* neurons (Fig. 4**a**b, **b**b). Pre-incubation of cells for 24 h with D4-Lnn restored the glutamate-induced calcium signaling back to control levels (from 2.50 ± 0.27, *N* = 7, *n* = 100, Fig. 4**a**b, **d**a to 1.43 ± 0.25, *n* = 7, *N* = 7, *p* = 0.0113, Fig. 4**a**c, **d**a after the use of D4-Lnn). Pre-incubation of cells with D4-Lnn interestingly abolished the KCL-induced opening of voltage-dependent calcium channels (from 1.65 ± 0.22, *N* = 9, *n* = 114, Fig. 4**a**b, **d**b to 0.30 ± 0.09, *N* = 5, *n* = 165, *p* = 0.0009, Fig. 4**a**(b, c), **d**b). ATP-induced calcium signal is significantly smaller in the SNCA x3 cells compared with control (CTRL: 1.92 ± 0.27, *N* = 4, *n* = 165, SNCA x3: 0.75 ± 0.11, *N* = 4, *n* = 114, *p* = 0.0062, Fig. 4**b**(a, c)). This was also restored by the pre-incubation of D4-Lnn (1.70 ± 0.30, *N* = 4, *n* = 120, *p* = 0.0251, Fig. 4**b**c, **d**c).

We previously showed that dopamine induces calcium signaling through dopamine receptor independent mechanisms [36, 37], and this includes (i) the opening of voltage-dependent calcium channels following dopamine uptake and depolarization of the plasmalemmal membrane and (ii) dopamine-induced lipid peroxidation and activation of phospholipase C and release of calcium from ER stores. Application of two stimuli of dopamine induces a typical calcium response in control cells [37]. However, *SNCA x3* cells exhibited a significantly higher calcium response to the second stimulus of dopamine compared with control cells (SNCA x3: from 0.61 ± 0.09, *n* = 160, *N* = 8, *n* = 160 to 1.89 ± 0.21, *N* = 6, *n* = 136, *p* = 0.0001; Fig. 4**c**(a, b)). The calcium response to dopamine in SNCA x3 was fully abolished when cells were pre-treated with D4-Lnn (0.16 ± 0.08, *N* = 4, *n* = 148, *p* = 0.0002; Fig. 4**c**c, **d**d). Complete prevention of the dopamine-induced calcium response by d-PUFAs is likely to be due to blockade of both insertion of channels into the membranes (in keeping with depolarizing stimuli), as well as blockade of the PLC/IP3 response. These data suggest that the incorporation of α-synuclein as a channel into the plasma membrane occurs when the PUFAs in membranes have undergone lipid peroxidation. Non-oxidizable PUFAs lead to membranes that are resistant to the insertion of oligomers and their channel-forming activity, and do not demonstrate glutamate/dopamine-induced calcium deregulation.

### Oligomer-induced toxicity is dependent on iron and lipid peroxidation

**Fig. 5:**
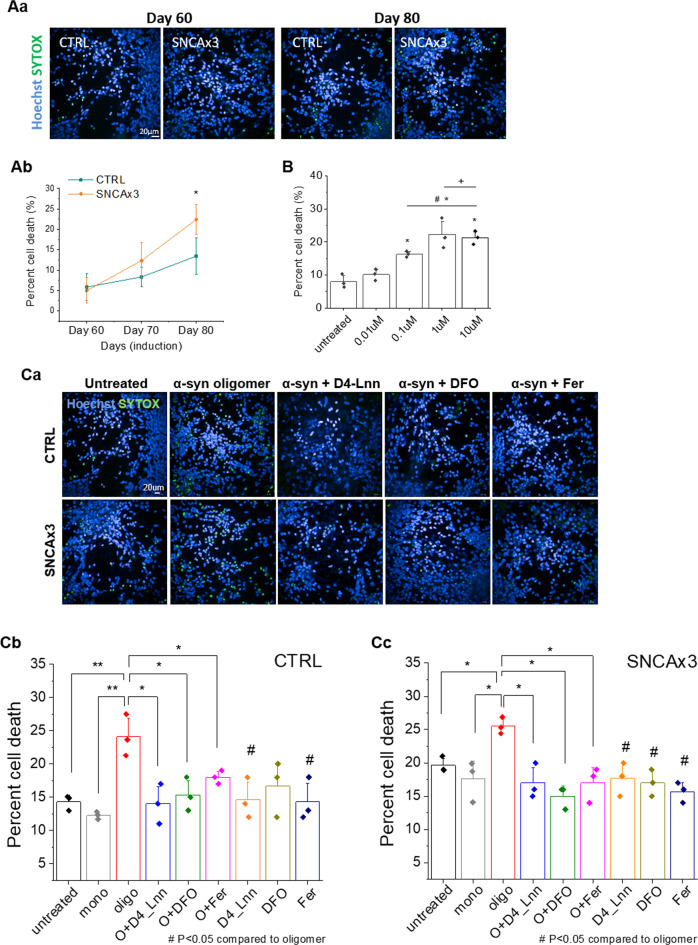
Increased cell death rate in SNCA x3 neurons is dependent on lipid peroxidation and on the presence of iron. **a** Cell viability of iPSC-derived neurons over time in culture. (a) Upon completion of neuronal maturation >day 60 [22], SNCA x3 cells show similar cell viability with CTRL. Neuronal loss of SNCA x3 is significantly increased over the following 20 days. (b) Representative images of cell death. **b** A dose response (0.01 μm—10 μm) effect of Erastin, a ferroptosis inducer, on toxicity in iPSC-derived cortical neurons. 100 μm Erastin data were excluded owing to the high toxicity preventing accurate quantification of cell death (*P < 0.05 compared with untreated, ^*#*^*P* < 0.05 compared with 0.01 μm and + *P* *<* 0.05 compared with 0.1 μm condition. *P < 0.05, ^#^*P* < 0.05 compared with untreated, ^+^*P* < 0.05 compared with 0.01 μm). **c** (a) Representative images depicting α-synuclein-induced toxicity induced by 1 μm α-synuclein monomer (10 nm oligomer) overnight and its rescue by blocking ferroptosis. d-PUFA (48 h), ferrostatin (1 h) and DFO (1 h) were pre-incubated prior to oligomer treatment). Blue-Hoechst 33342 (total number of cells); green-SYTOX green (dead cells). (b, c) Comparison of the effects of transition metal ion chelator (DFO), inhibitor of lipid peroxidation (D4-Lnn), and Fer (ferrostatin-1) a ferroptosis inhibitor on cell death, induced by α-synuclein oligomers within control (b, *N* = 3 per condition) and SNCA triplication (c, *N* = 3 per condision). *P < 0.05, **P < 0.001, ***P < 0.0001.

SNCA x3 cells exhibit reduced cell viability compared with CTRL over time in culture, shown in Figure. 5**a**(a, b). We tested the sensitivity to ferroptosis of our iPSC-derived synucleinopathy model using the ferroptosis inducer, erastin, and we show that erastin induces a dose-dependent increase in cell death, shown in Fig. 5**b**. Oligomer-induced oxidative stress is dependent on transition metal ions [17]. We tested the effects of metal ion chelator (Desferoxamine, DFO), inhibitor of lipid peroxidation (D4-Lnn), and a ferroptosis inhibitor (Ferrostatin-1) on cell death in synucleinopathy [38]. Application of oligomers but not monomers, induced cell death in control (Fig. 5**c**(a, b)) neurons and SNCA x3 (Fig. 5**c**(a, c)) neurons. Application of three different ferroptosis inhibitors, deuterated PUFAs, iron chelator DFO (Sigma), and ferrostatin-1 (Ferr-1, Sigma, Cat No. SML0583) each significantly reduced oligomer-induced cell death back to basal levels in control cells (Fig. 5**c**(a, b) and in SNCA x3 cells (Fig. 5**c**(a, c)). Taken together these data suggest that α-synuclein aggregates may induce ferroptosis, and that inhibitors of ferroptosis prevent α-synuclein-induced cell death, and inducers of ferroptosis exacerbate cell death.

## Discussion

Ferroptosis [39] describes a form of non-apoptotic regulated cell death occurring as a consequence of iron-dependent accumulation of lethal lipid peroxidation. Ferroptosis is characterized by cell swelling (oncosis), altered mitochondrial morphology [40], and unique features of lipid peroxidation with preferential oxidation of phosphatidylethanolamine [41]. Suppression of the formation of oxidized lipids halts cell death. The downstream pathways whereby lipid peroxidation leads to cell dysfunction or death are not fully established, but are proposed to include loss of membrane integrity, opening of pores and loss of ionic homeostasis, formation of free radicals that inactivates membrane-embedded proteins required for cell viability [42]. The major criteria for determining ferroptosis are the ability to suppress death by iron chelators, lipophilic antioxidants, inhibition of lipid peroxidation, and depletion of lipid peroxidation.

**Fig. 6:**
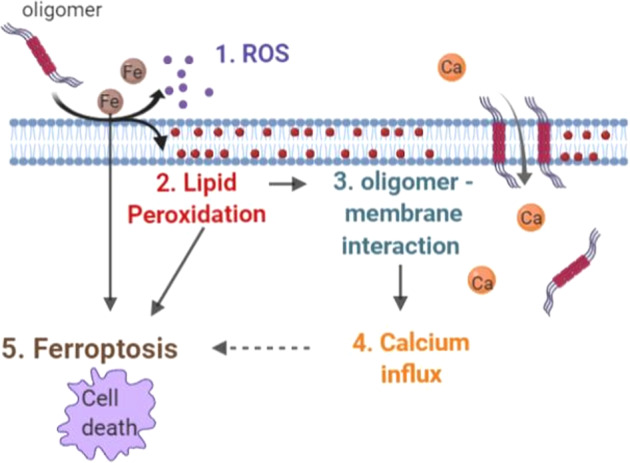
Schematic diagram illustrating mechanisms of α-synuclein-induced ferroptosis. Oligomeric α-synuclein induces (1) ROS and (2) lipid peroxidation within the membrane which results in an increase in (3) oligomer—membrane interaction, and consequently causes (4) calcium influx. The consequence of those events leads to cell death, (5) “Ferroptosis”.

In neurodegeneration [43], a number of ferroptosis features are commonly reported, in particular depletion of glutathione, accumulation of lipid peroxidation products, excess extracellular glutamate, decreased cortical GPX4, increased lipoxygenase (LOX) activity, protection from lipophilic antioxidant vitamin E (in AD), protection from iron chelators [44], and protection from pioglitazone, an ACSL4 inhibitor [45]. In addition, brain iron levels rise in during aging and neurodegenerative disease, which can be detected in living people [46] and in postmortem tissue. However, lipid peroxidation and ferroptosis has not been previously investigated in synucleinopathy models of PD [47].

Here, we study the complex intersection between protein aggregation, calcium deregulation and lipid peroxidation (Fig. 6). We have demonstrated that abnormal calcium fluxes, as well as abnormal intracellular stores, is an important biological property of specific α-synuclein oligomers, when applied exogenously to cell systems [13, 48]. In this study, we demonstrate that increased endogenous levels of α-synuclein oligomers, are associated with high cytosolic calcium influx in response to activation of glutamate receptors or changes in plasmalemmal membrane potential. This could be attributable either to high expression of potential-sensitive calcium channels or increased activation of glutamate receptors. Our data suggest an increased presence of potential-sensitive channels on the plasmalemmal membrane of the human neurons with high levels of oligomeric α-synuclein. Such channels are known to be formed by the effect of α-synuclein β-sheet-rich oligomers on membranes [13]. Notably, solution and solid-state NMR methods have confirmed that the exogenously applied oligomers used in this study, result in maximal membrane disruption by allowing the structured oligomer core to insert into the lipid bilayer and disrupt the integrity [14]. In our study, oligomers induce ion fluxes in the absence of the NMDA receptor components in artificial systems (liposomes), and additionally oligomers induce channel formation in the presence of NMDA blockade in outside-out patches in cells, Taken together, this supports the hypothesis that the α-synuclein oligomeric species alone are able to insert into membranes (in particular oxidized membranes), upon depolarizing stimuli, leading to a voltage-dependent increase in cytosolic calcium influx in response to glutamate, KCl, and dopamine.

The second key feature is the ability for oligomeric α-synuclein, both when applied exogenously or when generated endogenously, to induce the production of superoxide and hydrogen peroxide [17] and lipid peroxidation [49]. Notably, the mechanism of oligomer-induced ROS production and lipid peroxidation is iron-dependent, and non-enzymatic, likely dependent on the Fenton reaction [17, 18]. As protein aggregates interact with, and disrupt, lipid membranes, we sought to modulate this interaction through altering membrane properties by their oxidation state [50]. Lipidomics has previously been used to demonstrate that PUFAs are the most susceptible lipids in the course of ferroptosis [41], and that preventing peroxidation by supplementing cells with PUFAs deuterated at the susceptible bis-allcylic carbon suppresses ferroptosis [51]. Based on this, supplementation of media with deuterated D4-linolenic acid has shown to prevent lipid peroxidation [49], and also to prevent ferroptosis [51]. We show that supplementation of the media with deuterated PUFAs is able to prevent oligomer-induced lipid peroxidation. We further demonstrated that in the absence of lipid peroxidation, the α-synuclein-induced calcium dysregulation is also abolished, and physiological calcium signaling is restored. This effect is true for glutamate-induced, KCL-induced and also dopamine-induced calcium signaling, which induces both a calcium influx [36], as well as generates ROS [37]. We therefore confirmed that lipid peroxidation allows membranes to be more susceptible to aggregates inserting into them and disruption of ion fluxes. Stabilization of the oxidation state of the membrane prevents additional oligomeric channel insertion, and reduces ion fluxes across the membrane. Our hypothesis is that the oxidation state of the membrane confers its susceptibility to a physical and functional interaction with β-sheet-rich oligomeric proteins. It is possible that oxidized lipids recruit more oligomers to the membrane, or that any individual rare oligomer event results in greater disruption of the membrane and more calcium influx, and it is challenging to distinguish these two possibilities [52].

Iron chelators, d-PUFAs, and ferrostatin were all able to suppress cell death induced by toxic oligomeric α-synuclein, or by dopamine in neurons. Inhibition of toxicity using reduction of iron, suppression of lipid peroxidation, and ferrostatin meets the basic criteria set out for the definition of ferroptosis [53], and therefore we raise the hypothesis that α-synuclein may contribute to cell death by this pathway, whereas recognizing that other forms of apoptosis and necrosis also co-exist. The co-dependence of protein aggregation, membrane damage and oxidative stress in ferroptosis in neurodegeneration may underlie why models based on a single factor alone (e.g., GPX4 deletion) do not exhibit neurodegeneration [54].

Cellular aging, dopamine and α-synuclein oligomers place the cell in a state of aberrant ROS production and glutathione depletion, resulting in oxidative stress with lipid peroxidation. Oxidation of specific PUFAs, leads to the insertion of α-synuclein oligomers into the membrane forming channels that open in response to depolarization of the plasma membrane. This renders neurons vulnerable to physiological calcium signaling and exposes them to high levels of cytosolic calcium fluxes that may cause excitotoxicity and cell death. At least part of the α-synuclein aggregate-induced cell death is related to ferroptosis in which the iron-dependent accumulation of lipid peroxidation has an important role in the demise of neurons. While in cellular models, targeting lipid peroxidation specifically using deuterated PUFAs, is a useful tool to demonstrate the contribution of lipid oxidation to calcium fluxes, its direct application to patients may be hindered by the important physiological roles that lipid oxidation also has in neuronal function. Nonetheless, modulation of lipid peroxidation or ferroptosis may represent new potential therapeutic approaches for PD.

## Supplementary information

Supplementary figure legends

Supplementary Figure 1

Supplementary Figure 2

Supplementary Figure 3

Supplementary Figure 4

Supplementary Figure 5

Supplementary Figure 6

Supplementary Figure 7
