# Supplementary material for: Alpha synuclein aggregation drives ferroptosis: an interplay of iron, calcium and lipid peroxidation
Source: Cell Death Differ. 2020 Apr 27;27(10):2781–96. doi: 10.1038/s41418-020-0542-z (PMC7492459; doi:10.1038/s41418-020-0542-z)
Supplement: Supplementary file 1 — Supplementary figure legends [file 41418_2020_542_MOESM1_ESM.docx]

**Supplementary Figure legends**

**Supplementary Figure 1. Characterization of ­­aggregates using single molecule TIRF imaging.** Aa) sm-TIRF of α-synuclein aggregation time points in the presence of 5 μM of ThT. From left to right 0h (monomers only, no ThT signal), 8h (fluorescent puncta indicates the presence of oligomers – red circles), and 24h (most αS has been converted into fibrils). Images were taken using 2.8 µM of α-synuclein and are displayed with the same contrast except for 24h. Error bar is­ 5 µm. Ab). FRET image of T29h after incubation of α-synAF-594 + α-synAF488. Representative image of early aggregates (16). sm-TIRF FRET image of T72h after incubation of aSYn-594 + aSyn-488. In this study, we used T29h incubation samples as oligomeric species. Scale bar is­ 5μm.

**Supplementary Figure 2. Characterization of synucleinopathy model** Aa) Representative images of α-synuclein immunocytochemistry using total α-synuclein antibody. Ab) Monomeric α-synuclein expression is significantly higher in SNCA x3 cells than control, N = 4 CTRL & SNCAx3. The fluorescent intensity (arb.U) of total α-syn antibody expression was calculated using Image J software. The same threshold to subtract background was applied to all images. Average intensity per cell was compared. α-syn: total α-synuclein antibody (MJFR1, Abcam). **P<0.05, **P<0.001, ***P<0.0001.*

**Supplementary Figure 3. Alteration in calcium stores in iPSC-derived neurons with SNCA x3.** Cells loaded with Fura-2 were first treated with an inhibitor of SERCA (1μM Thapsigargin) to deplete Ca2+ from the ER in Ca2+free medium – the change in cytosolic calcium signal in response to Thapsigargin was taken as the ER pool. Subsequent application of ionomycin eliminates any remaining [Ca2+] gradient between all membranes. Following Thapsigargin, the remaining gradient is largely due to the mitochondria-cytosolic [Ca2+]. Therefore the change in cytosolic calcium following Ionomycin can be taken to represent the mitochondrial Ca2+ pool. Aa) The ER Ca2+ pool in neurons with SNCA x3 was 2.9-fold smaller than in control neurons (1.41±0.25 Fura-2 ratio, n=33 for control; 0.51±0.03, n=30 for SNCA x3; p<0.05), in contrast, the mitochondrial calcium pool was significantly higher (3.39±0.09 Fura-2 ratio (n=30) compared to 1.86±0.21 in control (n=33); p<0.05. Ab) The mean values of the calcium stores are shown in the histogram (Mean ± SEM). **P<0.05, **P<0.001, ***P<0.0001.*

**Supplementary Figure 4. Abnormal calcium signaling in hES-derived neurons with overexpression of α-synuclein.** Both control (Aa) and α-synuclein o/e (Ab) cells generated action potentials of classical shape in response to current injection, which could be blocked by Tetrodotoxin (1µM TTX). B) 10µM Glutamate (Ba) or 50 mM KCl (Bb) induced calcium signals in α-synuclein overexpressing cells was significantly higher than those found in control cells. Bb) Non-neuronal cells overexpressing α-synuclein demonstrated a higher ATP-induced (100µM ATP) calcium signal than control cells. Bd) Histogram graph demonstrating a higher response to KCl and ATP in α-synuclein overexpression cells. **P<0.05, **P<0.001.*

**Supplementary Figure 5. Measuring α-synuclein oligomer concentration by ELISA.** A) α-synuclein oligomer is not detected in cells treated with a range of concentrations of human recombinant monomer (50nM - 2uM) by a commercially available α-synuclein oligomer ELISA kit (CSB-E18033h, Generon), confirming its specificity for aggregates of α-synuclein. B) Lysates from SNCA x3 cells have a higher α-synuclein oligomer concentration using a ELISA detecting oligomer concentration, N = 3 CTRL & SNCA x3.

**Supplementary Figure 6. Abnormal ROS production in hES cells with overexpression of α-synuclein.** ROS production is higher in hES derived neurons with α-synuclein overexpression (SNCA o/e) in (Aa) and the representative trace of generation of ROS in CTRL and SNCA o/e cells in (Ab). ****P<0.0001.*

**Supplementary Figure 7. Characterization of α-synuclein – membrane interaction and uptake, dependent on the oxidative status of the membrane.** Aa) Representative images of exogenous oligomeric α-synuclein uptake (AF488 fluorescence, green) into the cytoplasm (membrane labelled with red) in control neurons; Upper panel: AF488 uptake in control condition and Lower panel: after overnight pre-treatment with Trolox. Ab) Oligomer uptake was significantly reduced when membrane oxidation was inhibited by Trolox, N = 5 basal & Trolox. **P<0.05.*
