# Supplementary figures and images for: Alpha synuclein aggregation drives ferroptosis: an interplay of iron, calcium and lipid peroxidation
Source: Cell Death Differ. 2020 Apr 27;27(10):2781–96. doi: 10.1038/s41418-020-0542-z (PMC7492459; doi:10.1038/s41418-020-0542-z)

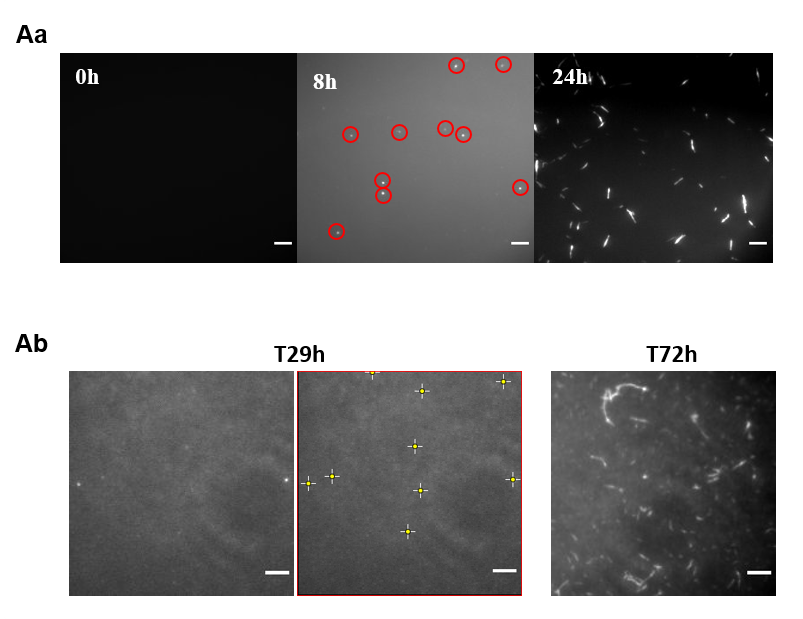

Supplement: Supplementary file 2 — Supplementary Figure 1 [file 41418_2020_542_MOESM2_ESM.tif]

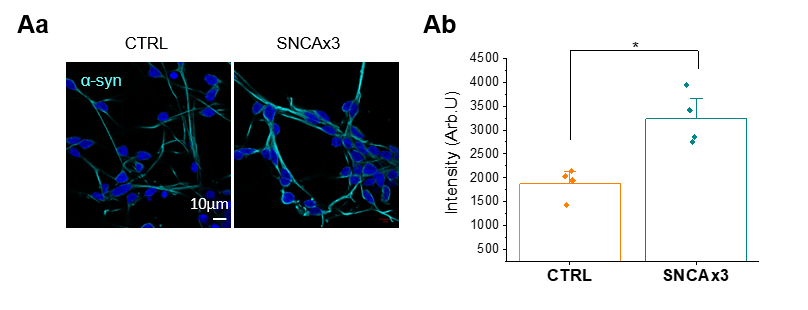

Supplement: Supplementary file 3 — Supplementary Figure 2 [file 41418_2020_542_MOESM3_ESM.tif]

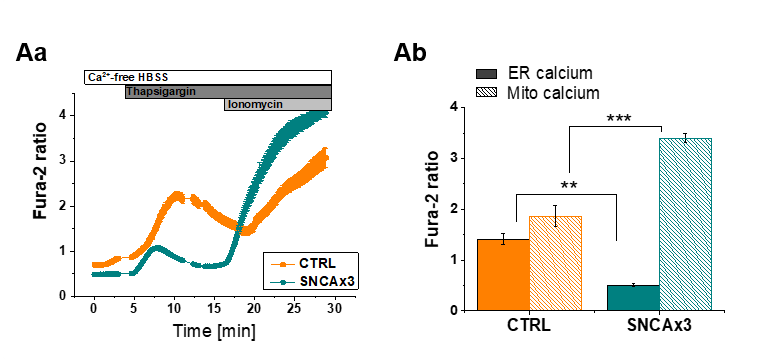

Supplement: Supplementary file 4 — Supplementary Figure 3 [file 41418_2020_542_MOESM4_ESM.tif]

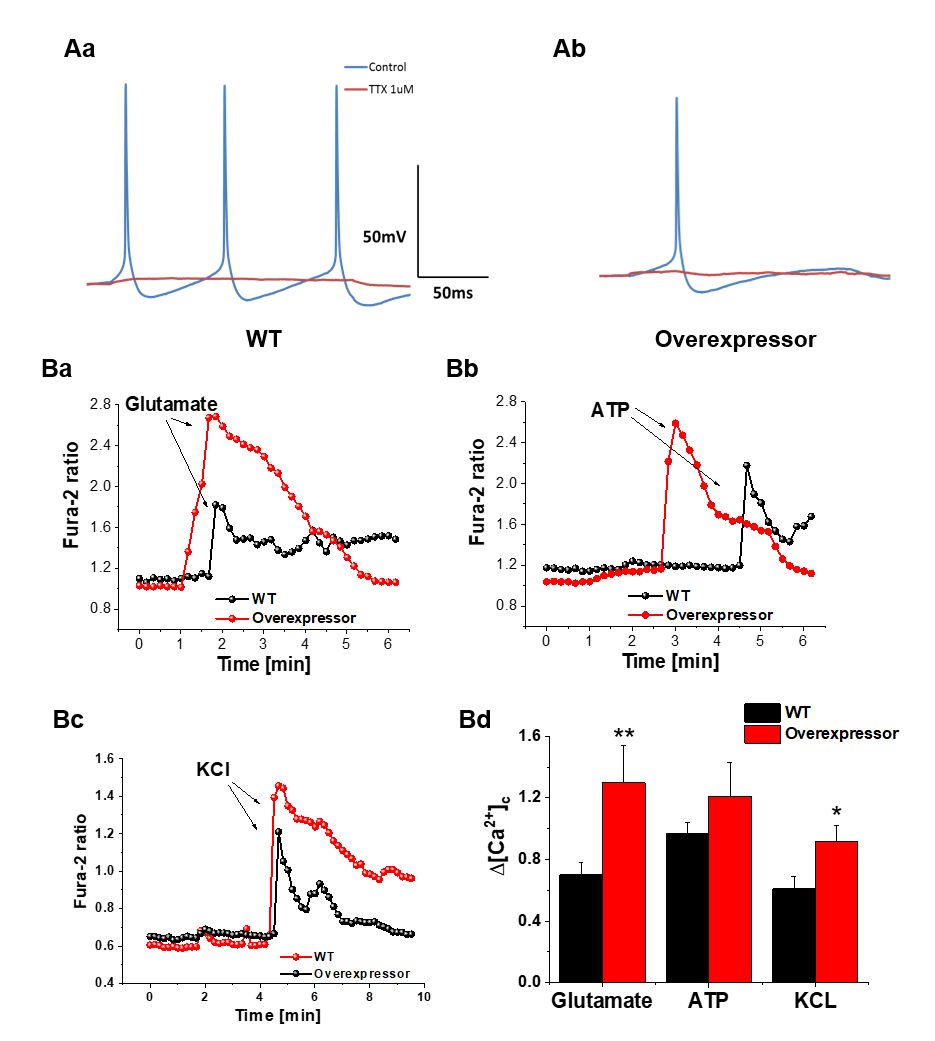

Supplement: Supplementary file 5 — Supplementary Figure 4 [file 41418_2020_542_MOESM5_ESM.tif]

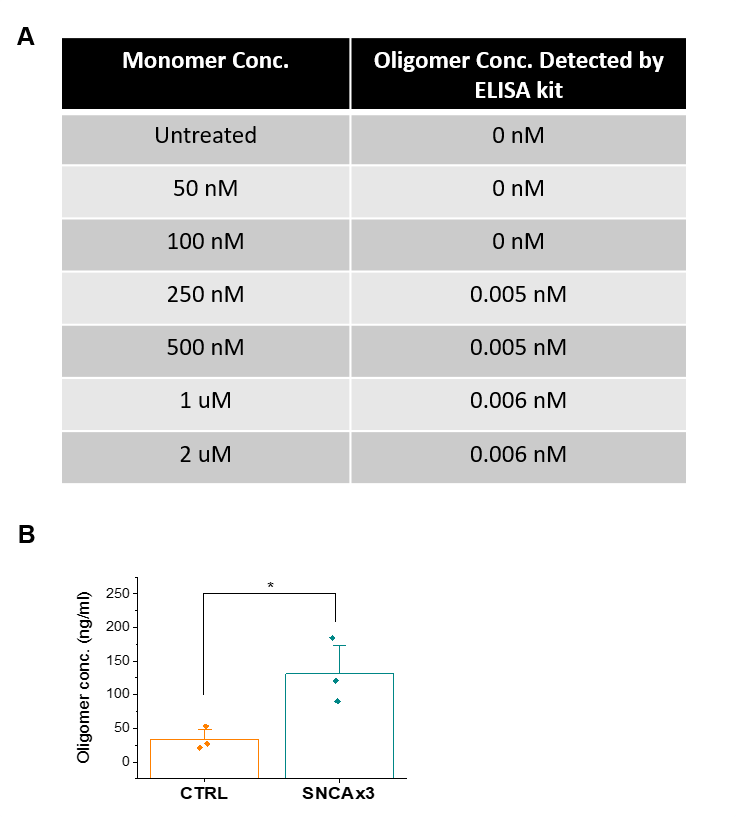

Supplement: Supplementary file 6 — Supplementary Figure 5 [file 41418_2020_542_MOESM6_ESM.tif]

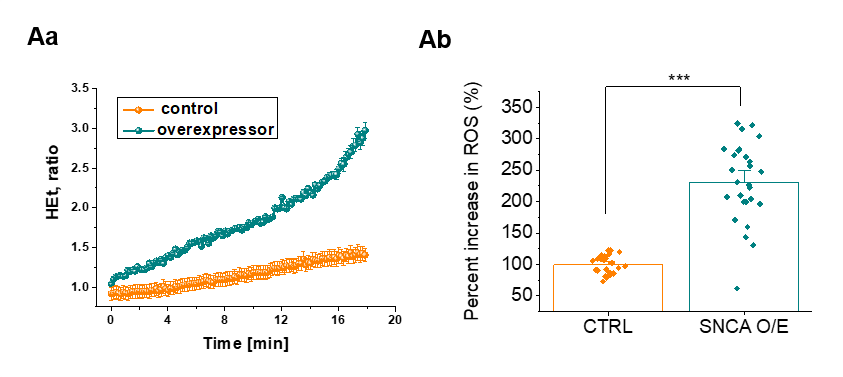

Supplement: Supplementary file 7 — Supplementary Figure 6 [file 41418_2020_542_MOESM7_ESM.tif]

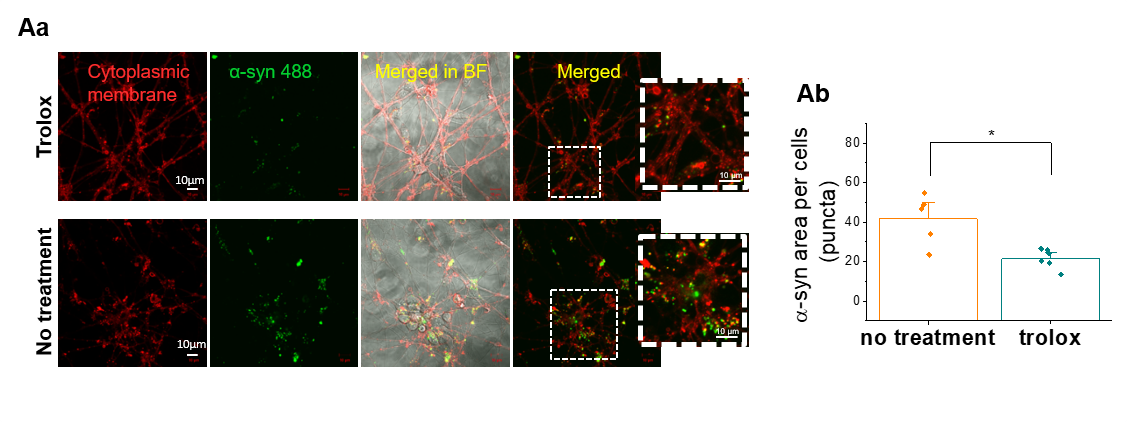

Supplement: Supplementary file 8 — Supplementary Figure 7 [file 41418_2020_542_MOESM8_ESM.tif]
